# Nanodecoys: A Quintessential Candidate to Augment Theranostic Applications for a Plethora of Diseases

**DOI:** 10.3390/pharmaceutics15010073

**Published:** 2022-12-26

**Authors:** Sampreeti Chatterjee, Karthick Harini, Agnishwar Girigoswami, Moupriya Nag, Dibyajit Lahiri, Koyeli Girigoswami

**Affiliations:** 1 Department of Biotechnology, University of Engineering & Management, Kolkata 700160, West Bengal, India; 2 Medical Bionanotechnology, Faculty of Allied Health Sciences, Chettinad Hospital and Research Institute, Chettinad Academy of Research and Education, Kelambakkam 603103, Tamil Nadu, India

**Keywords:** nanodecoys, biomimetic cells, ghost cells, cell membrane coating, nanotheranostics

## Abstract

Nanoparticles (NPs) designed for various theranostic purposes have hugely impacted scientific research in the field of biomedicine, bringing forth hopes of a future revolutionized area called nanomedicine. A budding advancement in this area is the conjugation of various cell membranes onto nanoparticles to develop biomimetic cells called ‘Nanodecoys’ (NDs), which can imitate the functioning of natural cells. This technology of coating cell membranes on NPs has enhanced the working capabilities of nano-based techniques by initiating effective navigation within the bodily system. Due to the presence of multiple functional moieties, nanoparticles coated with cell membranes hold the ability to interact with complex biological microenvironments inside the body with ease. Although developed with the initial motive to increase the time of circulation in the bloodstream and stability by coating membranes of red blood cells, it has further outstretched a wide range of cell lines, such as mesenchymal stem cells, beta cells, thrombocytes, white blood cells, and cancer cells. Thus, these cells and the versatile properties they bring along with them open up a brand-new domain in the biomedical industry where different formulations of nanoparticles can be used in appropriate dosages to treat a plethora of diseases. This review comprises recent investigations of nanodecoys in biomedical applications.

## 1. Introduction

For the last couple of decades, nanoparticles ranging in size between 1 to 1000 nm have been intensively studied and worked on in drug delivery mechanisms. Nanoparticles have shown immense potential in the field of biomedical sciences due to their vivid physiochemical properties, easily manipulable properties, and structures [1]. The advantages of nanoparticles are their high loading capacity, tunable physiochemical properties, solubility, stability, in vivo behavior, ability of specific targeting, and increased efficacy. Nanoparticles have an extended circulation life, and they remain in the system for a longer time than directly administered drugs [2]. By nanosizing a formulation, the dissolution rate of the drug can be increased, leading to the enhancement of the absorption levels of the drug and its bioavailability. Improved tissue selectivity was achieved by using nanoparticles and they can be used to enhance protection. They are also used in renal clearance of drugs that are readily degraded or have short half-lives, such as small peptides and nucleic acids, for pharmacological effects [3].

**Figure 1:**
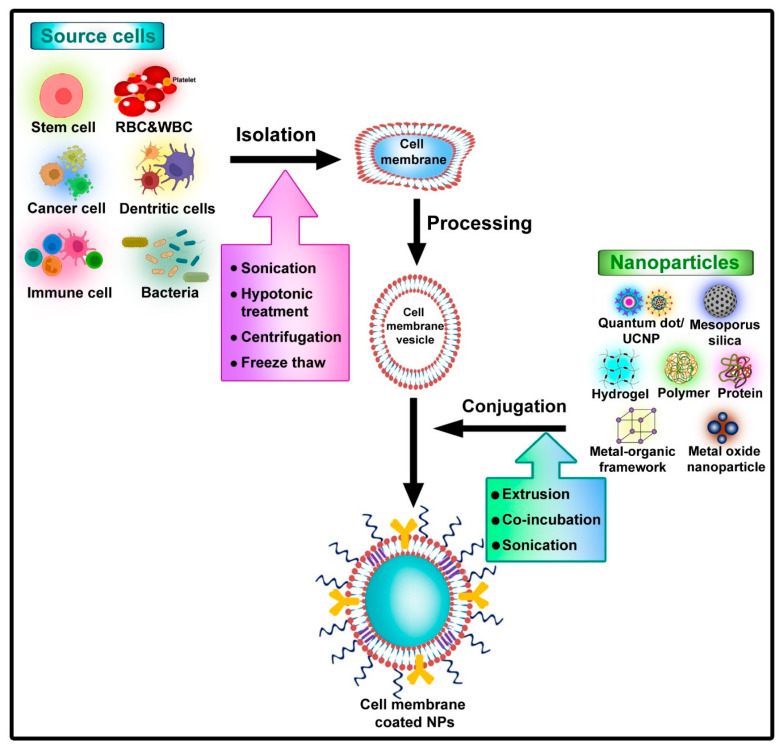
Method of fabrication of nanodecoys.

In most scenarios, directly administered drugs have a very short lifespan. Due to the uneven distribution of the drug component in the blood, they are very difficult to exterminate from the body system. The non-uniform distribution also leads to low targeting efficiency levels. Improper distribution of the drug may also cause unwanted adverse reactions in other cells of the body by tampering with their normal functioning. To counter these shortcomings, nano-drug delivery systems have been developed. Nanoparticles work as brilliant carriers of drugs, genes, or vaccines. They are extremely biocompatible and work well in a suitable biosystem. All the properties of the outcomes also majorly depend on the method chosen for the preparation (Figure 1). Due to their high specificity and targeting efficiency, comparatively lower amounts of the drug are incorporated into the living system, thus reducing the toxicity level [4].

## 2. Toxicological Aspects of Nano-Based Drug Delivery System

Just like a coin has two sides, even nanoparticles have their equal share of advantages and disadvantages. The usage of nanoparticles has eased the drug delivery system considerably by adding desirable features such as long circulation life, specific targeting system, and toxicity reduction. However, nanoparticles have certain limitations, which curtail their clinical abilities. Most nanoparticles are unable to overpower the body’s immune response. Surface functionalization, such as PEGylation of nanoparticles, is performed to reduce the susceptibility in elimination via the reticuloendothelial system (RES). Various studies have identified that repetitive use of PEGylated nanoparticles induces an immune response in the body, leading to faster elimination of nanoparticles and curbing their translation. The favored targeting capacity of nanoparticles depends on the modification of the surface, which is challenging to fabricate and formulate [5].

The existing delivery system contributes significantly to in vivo applications. However, nanosystems still suffer barriers in the delivery system due to obstructions placed by the immune system, biological adhesion, and specific site targeting. The PEGylation or addition of phospholipid modifiers has been shown to be helpful in extending the time of circulation due to their high hydrophilicity. These simple modifications can readily inhibit the bio-adhesion of blood constituents and also inhibits RES uptake. Despite the successful implementation of surface modifications, synthetic nano-based systems tend to induce an adverse immune response and accelerate rapid clearance. Therefore, it is necessary to develop a bio-surfacing approach to improve current synthetic nanosystems, which enables prolonged circulation time to provide an efficient therapeutic effect [6].

## 3. Cell Membrane-Coated Nanocarrier System

Cells communicate with the environment they thrive in and other neighboring cells via a very integral structure called a cell membrane. Cell membranes, the outermost layer of a cell, is made up of a dual layer of lipoproteins. This lipoproteinaceous layer functions in keeping the contents of the cell separated from the surrounding matter. Its exterior part is hydrophilic, and the interior parts are hydrophobic in nature. The cell membrane ensures the interaction between the cell cytoplasm and the surrounding environment. Cells require a selective range of nutrients for their proper functioning. They also require a set amount of water and the elimination of toxic materials from it. This happens efficiently due to the semi-permeability of the membrane. They can carry out selective intake and excretion of different materials. Cell membranes also provide a platform for cell recognition markers that are genetically unique. These markers can guide the cell to identify the difference between a foreign material and known material. This property aids the immune system of the cell/organism to build a defense system and helps to maintain the cellular environment. The cell membrane has the ability to form encapsulated vesicles, which work as physical barriers between the core and surroundings of the vesicles. These may be used to design carriers for the delivery of drugs and also act as a template for the synthesis of nanoparticles. Source cells can be processed to isolate the cell membrane with functional groups on the surface and empty the interior contents by making a hollow vesicle. These types of hollow cells without a nucleus are named Ghost cells. Desired molecules can pass through the cell membrane while unwanted molecules cannot, which can serve as a nanoreactor to permit substrates to travel in and out of the cell membrane. They also prevent the interior vesicular enzymes from denaturation [7].

Another integral function of the cell membrane is cellular communication during various biological processes, which occurs by transmitting and receiving information. Cells contain unique surface molecules like receptors that act as signatures, which enable cellular recognition, migration, activation, and several other such functions. Red blood cells, for instance, have a circulation period of approximately 120 days. Biomolecules interact with specific cells by binding to the receptors found on the membrane through the ligand–receptor binding mechanism (lock and key mechanism). Thus, it can be inferred that these biomolecules can in turn communicate with cell membrane-coated nanoparticles (CM-NP) by binding to the same specific receptors [6].

One of the major targets that researchers aim to achieve in this field of nanomedicine is the effective and efficient targeting of a drug at a specific site and proper binding in vivo. A longer circulation period is the most necessary feature that is considered while developing a nanomedicine to ensure appropriate targeting (active or passive delivery). Surface modification is the most advantageous conception of a range of nanoparticles, which enhances their circulating performance in vivo. The cell membrane-coating strategy enables the nanoparticle-based drug delivery system to circulate in the bloodstream without any hindrances. Compared to artificially synthesized vesicles composed of lipid bilayers, naturally derived cell membrane-based vesicles consist of several membrane-related surface functional groups, such as proteins, carbohydrates, and antigens. This function protects the cell, prevents biofouling, and instigates specific recognition and intracellular communications. Recently, several natural cells such as erythrocytes, thrombocytes, stem cells, cancer cells, macrophages, and bacterial cells (*E. coli*) (Table 1) served as the vital source for the extraction of the cell membrane and constructed versatile functioning bio-hybrid delivery systems through the bottom-up approach (self-assembly) [6,8].

### 3.1. Erythrocytes Coated Nanodecoys (ENDs)

Natural red blood cells (RBC) or erythrocytes are ideal selections for cell membrane extraction to create biomimetic cells. A major justification of the above statement is the suitable structural composition, as they lack a nucleus, ribosomes, and mitochondria. This makes extraction of the cell membrane much easier. RBCs are abundant in the body since their key function is to supply oxygen to every cell of the body. Due to this, RBCs have free clearance from the immune system and can easily pass throughout the cardiovascular system and also to all the organs [9,10].

Human erythrocytes are homologous to the autologous immune cells. This allows them an easy clearance from the body’s immune system and they are cleared out before the end of their life span by the spleen, only when they are damaged or malformed. RBCs have a circulation life of up to 120 days. These features make them ideal for carrying nanoparticles in vivo, allowing them to escape the immune system. Research has shown that the membranes of RBCs are enriched with self-markers, which aid the nanoparticles in remaining camouflaged from the immune system. This greatly enhances the durability of poly (lactic-co-glycolic acid) nanoparticles from a few hours straight up to 40 h [2,11].

A vast range of membrane proteins is the primary mediator behind the long-term circulatory efficiency of erythrocytes. CD47-membrane protein is an integral protein having five membrane-spanning zones and is deeply seated in the RBC membrane alongside an IgV-like extracellular domain. This ensures the survival of the RBCs in circulation. CD47 also prevents the uptake of erythrocytes by macrophages via sending signal and acts as self-markers. Particularly speaking, the signal-regulatory protein, alpha (SIRPα) glycoprotein expressed by phagocytes, comes into contact and identifies the CD47 as a signal preventing the immune cells from “eating” or engulfing the RBCs. Other membrane proteins, including C8 binding protein (C8bp), decay accelerating factor (DAF), homologous restriction protein (HRP), membrane cofactor protein (MCP), complementary receptor 1 (CR1), and CD59 on the surface of RBC membranes, also work as a defense against attacks and engulfs. These membrane proteins can decrease the effect of immune response by posing the nano-based drug carriers as “self” cells, helping them attain longer circulation life. A research group studied the half-life of PEGylated nanoparticles and RBC-NP, which was found to be 15.8 h and 39.6 h, respectively. This result indicated that the RBC-NP provided a prolonged circulation of nanoparticles in the bloodstream than biocompatible polymer coating [11].

RBC membranes, otherwise called RBC ghost cells, are derived by treating whole blood. A protocol reported by Li et al. included the collection of whole blood from an animal model (in this instance, ICR mice aged six weeks) centrifugation at 4 °C in RCF (Relative centrifugal force) of 700× *g* for 10 min for the removal of thrombocytes, white blood cells (WBC), and plasma. Further treatment, purification, and storage of the resultant ghost cells were well explained [2].

Extraction of RBC membrane via hypotonic treatment was first reported in 2011 by Hu et al. [10]. Later, after further studies and research, red blood cells self-enriched with oxygen were designed, which enhanced the circulation period. It was observed via transmission electron microscopy that RBC ghost cells can coat nanoparticles of 65–340 nm diameters. Along with that, due to the presence of surface modifiers, they even protected and stabilized the payload [9].

### 3.2. Leukocytes Coated Nanodecoys (LNDs)

White blood cells (WBC) or leukocytes are classified into several sub-categories based on the varied range of functions they serve—monocytes, mast cells, macrophages, lymphocytes, granulocytes, eosinophil, neutrophils, and basophils. WBCs are present abundantly in the body and play integral roles in immune mechanisms. White blood cells have a very complex build, comprising several complicated nuclei and cell organelles, which makes the extraction of ghost cells difficult.

Despite the difficulty faced in extracting the ghost cell, WBCs still stand as an optimum choice for creating biomimetic cells due to their unique properties and features. When there is an infection, injury, or entry of microorganisms, leukocytes reach the site of action via the bloodstream to cause inflammation and secrete various cytokines. Leukocytes are also known to have chemotactic properties. Tumor tissue has a characteristic to induce a chronic inflammatory response. This creates a synergy between tumor tissues and the subtypes of leukocytes. This is why WBC membrane-coated nanoparticles are significantly being studied for cancer drug delivery systems [12].

For instance, porous silicon nanoparticles coated with WBC membranes with a high sucrose density were formulated via a purification process. These nanoparticles were then conjugated with APTES (positive (3-Aminopropyl) triethoxysilane), for stabilizing the membrane coating. The coating on these nanoparticles made them less susceptible to antibody opsonization and adsorption by serum proteins than the untreated nanoparticles [9]. Wang et al. synthesized a drug carrier system to deliver an anticancer agent—doxorubicin. The method involved the synthesis of gallium nanoswimmers via the pressure-filter template technique. The physical extrusion method was performed for the extraction of WBC membrane, which was then fused onto the nanoparticle through an acoustic-assisted nanovesicle fusion procedure and loaded with the drug. The synthesized nanovesicle was characterized using various photophysical tools. The drug loading capacity was found to be about 13.1% wt. The drug release profile studies revealed that the vesicle synthesized can perform pH-responsive release by comparing the nanoparticle with and without WBC membrane coating [13]. Li et al. fabricated glycyrrhetinic acid nanoparticles loaded on the WBC membrane coated with PLGA (poly (lactic-co-glycolic acid)). The particle was synthesized aiming to induce cell apoptosis and provide immunotherapy against solid malignancies. The in vivo study showed improved T-cell immune response against colorectal tumors and leukemia. This study concluded that the synergistic effect of glycyrrhetinic acid and ferrotherapy could provide a superior therapeutic effect [14]. A recent research investigation done by Wang et al. reported that monocytes, alongside appropriate drugs, provided synergistic immuno-chemotherapy. As for the drug, rapamycin was chosen, and PLGA nanoparticles were used for the encapsulation of the drug through the nanoprecipitation method. Further co-extrusion and sonication were performed to coat the membrane of monocytes. The results obtained from the in vivo and in vitro experiments showed that the formulation prepared achieved binding to the inflammatory epithelial cells to inhibit the proliferation of inflammation in the injury site. Thus, the particle contributed immunotherapy against the ischemic stroke-induced rat model, and also enhanced the regeneration [15]. Macrophages are specialized innate immune cells responsible for maintaining the physiological ambiance. Macrophages are now being developed to carry nanotherapeutics to treat various disorders, especially cancer, as they can target, bind, and deliver the payload, thereby increasing the biocompatibility of type carriers. A review report by Xia et al. discussed the advantages of macrophage-coated nanocarriers in drug delivery and cancer therapy. That article also complies with the recent research evidence that proves that macrophage-coated nanoparticles provide a better therapeutic effect [16]. Lymphocytes are also being widely studied to encapsulate nanocarriers. Mühlberger et al. worked with T-cell lymphocytes and designed citrate-coated superparamagnetic iron oxide nanoparticles (SPIONs) co-encapsulated with T-cells for treatment against solid tumors. The authors performed an alkaline coprecipitation method for the preparation of SPIONs and an in-situ technique for the coating of citrate. The in vitro experiment conducted showed that the T-cells can be controlled by the magnetic field even after the loading of citrate-coated SPIONs. This preliminary study concluded that the formulation designed can be further investigated to develop into a fully operational immuno-therapeutic procedure [17]. Alveiri et al. recently studied the granulocytes-coated SPION as a diagnostic agent for inflammations and other infections. All the experiments were carried out in vitro, where magnetic resonance imaging (MRI) and near-infrared fluorescence (NIRF) imaging were used to test the agent. The result implies that the formulation can be used to detect inflammation/infections. This study also found that Percoll with Ficoll was the best isolation technique to isolate granulocytes for human peripheral blood [18].

### 3.3. Thrombocytes Coated Nanodecoys (TNDs)

Thrombocytes or platelets are oval disc-shaped small fragments of cytoplasm formed from matured megakaryocytes. Thrombocytes are found in the body at a high density of 150,000–350,000 cells per microliter. Their primary function is to protect the integrity of vasculature. Detailed studies have shown that this hemostatic property of thrombocytes is important in several ways in promoting cancer’s metastatic progression. This is due to the close interactions between tumor cells and thrombocyte cells. Since then, thrombocyte cells have been considered an optimum choice for the production of biomimetic cells in drug delivery. Pure thrombocytes have low antigen levels in them, and thus, are less prone to immunogenic responses [19]. Hu et al. formulated a thrombocyte membrane-based nanocarrier conjugated with ligand (TRAIL) that can induce TNF (tumor necrosis factor)-related apoptosis and encapsulated it with doxorubicin. The in vivo analysis demonstrated that the vehicle showed strong antitumor efficacy in both cases of subcutaneous tumor and the metastatic stage [20]. The strong cytoskeleton maintains the integrity and structure of thrombocytes during circulation. However, thrombocytes get activated when they come across external stimuli. The shape of the cells undergoes a drastic change, turning into a spiny sphere-like structure from a smooth disc-like structure. Influxes of calcium are the primary initiator of the deformation process, instigating the formation of spike-like microfilaments. These microfilaments can secrete coagulation or an adhesive protein that entangles the surrounding thrombocytes and forms plugs those seals the damaged blood vessels. This type of unique shape creates an interaction between the cells and their environment. In the case of circulating tumor cells, they typically choose thrombocytes to protect them from barriers and capture immune cells. They also contribute to the surface aggregation of tumor cells in motion. These qualities of thrombocytes inspired researchers to work on a thrombocyte-based delivery system, which could readily improve therapeutic performance [21].

### 3.4. Stem Cell Nanodecoys (SCNDs)

Stem cells (SCs) can differentiate into a vast range of cells and can transform into any cell type of the body. SCs have shown tendencies of tumor targeting via chemotaxis and surface interaction. MSC (mesenchymal stem cells) or stromal stem cells are the most preferred type of stem cells in research. Researchers have attempted to use stem cells as cargo carriers for targeted delivery to tumor cells in order to attain better therapeutic efficiency. However, the greatest threat that stem cells pose is their ability to differentiate into any type of cells. Any sudden transformation into an unwanted cell type in the presence of a complicated tumor environment may lead to a significant setback in the therapeutic procedure. This drawback can be tackled by the usage of MSCs specifically. Mesenchymal stem cell membrane-coated nanomaterials possess extremely accurate tumor targeting capability due to the outer membrane coating, as it lessens the risk of cell differentiation. Induced pluripotent stem cells (iPS), recently produced by Liu et al. from adult cells, contributed to the development of stem cell membrane-based nanotherapeutics [1].

MSCs are pluripotent progenitor cells capable of self-renewal, used in a variety of biomedical applications that emphasize tissue engineering/regenerative medicine. MSCs are also used in chemotherapy and to reduce the side effects of treatment against autoimmune diseases. Coating nanoparticles using these cells’ membranes makes it possible to produce a nanocarrier system with similar orientational functions. In recent years, the role of MSCs in the field of tumor initiation, metastasis, and carrying of antineoplastic agents to the target cell is being studied. Gao et al. used the membrane of MSCs derived from bone marrow to coat doxorubicin and gelatin nanogel. This construct showed high stability and pH-responsive release behavior with high loading capacity. The cytotoxicity was found to be lesser than naked nanoparticles and free drugs. They exhibited highly specific accumulation in the diseased site [22]. Table 1 summarizes the important nanodecoys used in biomedicine.

**Table 1:** Recent Advances in Nanodecoys for Biomedical Applications.

S. No	Cell Membrane/Extraction and Coating Method	Nanoparticles	Surface Modifications	Drugs	Target Cell/Disease/Pathogen	Applications/Functions and Limitations	Key Features	References
1.	Macrophage. The extrusion technique was used to coat macrophage membranes on gold–silver nanocages in order to fabricate macrophage-membrane-coated nanoparticles.	Gold/Silver nanocages	-	Rhodamine B	Osteomyelitis and local infection	Anti-bacterial photothermal therapy. Using macrophage membranes coated with bacterial pretreatment, this nanosystem can be used for precision/personalized medicine. The unique construction of gold-silver nanocages (hollow interiors and porous walls) makes it possible to load antibacterial drugs within these nanosystems for on-demand controlled release under NIR light. Limitations exist for the clearance of metal nanoparticles from our bodies.	Improved the bactericidal effect upon irradiation of NIR	[23]
2.	Erythrocytes. In order to prepare human RBC nanosponges (hNS), three steps were taken: (i) hypotonic treatment of packed hRBCs to obtain RBC membranes, (ii) nanoprecipitation by adding poly(lactic-co-glycolic) acid (PLGA) in organic solvents to an aqueous phase to prepare polymeric cores, and (iii) sonication of hRBC vesicles onto PLGA cores.	Polymeric nanoparticles	-	-	Hemolytic toxins	Neutralizing the effectiveness of pore-forming toxins (PFTs). hNS was tested against four representative PFTs (melittin, listeriolysin O, α-hemolysin, and streptolysin O) in vitro and in vivo for its capacity to absorb and neutralize these toxins. Limitations of this study involve the risk of blood-borne diseases if the isolation process of the erythrocyte is compromised. Scaling up human erythrocyte-derived membranes has ethical issues.	The nanosponges possessed novel antivirulence applications against hemolytic toxins of various strains of bacteria	[24]
3.	Neutrophil. For the synthesis of neutrophil-NPs, purified and activated human peripheral blood neutrophil plasma membrane was coated onto poly(lactic-co-glycolic acid) (PLGA) polymeric cores.	PLGA	-	-	Rheumatoid arthritis	Anti-inflammatory strategy. Their prophylactic regimen was used to test the effectiveness of neutrophil nanoparticles in treating early-stage arthritis in CIA mice. The limitation of this study is the scaling up of neutrophil-derived membranes and manufacturing issues.	The particle neutralized the proinflammatory cytokines, targeted the cartilage matrix, and suppressed the severity of arthritis	[25]
4.	Platelet. A repeated freeze-thaw process was used to extract platelet membrane from platelet rich plasma (PRP). Nanoprecipitation was used to prepare the PLGA cores. PLGA nanoparticles (PNP) were prepared by mixing the nanoparticles with PEGylated platelet membrane and sonicating them. PNP loaded with rapamycin (RAP-PNP) was prepared using the same method except that 800 mg of rapamycin was added to the PLGA solution.	PLGA	-	Rapamycin	Atherosclerosis	Targeted drug delivery. By mimicking platelets’ inherent adhesion to atherosclerosis plaques, poly(DL-lactide-co-glycolide) nanoparticles (PNP) were explored as a drug delivery system targeting atherosclerosis plaques using the immunosuppressant Rapamycin (RAP). In apolipoprotein E-deficient (ApoE-/-) mice, PNP encapsulating RAP (RAP-PNP) was tested for anti-atherosclerosis activity against atherosclerotic plaques both in vitro and in vivo. The limitation of this study was that the membrane is human-derived, which can have ethical concerns. Moreover, it induces macrophage autophagy, which may interfere with normal homeostasis.	Target and delay atherosclerotic plaques. A promising platform for the treatment of atherosclerosis	[26]
5.	Cancer cell. Adenocarcinoma cells (MCF-7) were sonicated in buffer solution with protease inhibitor cocktail and differentially centrifuged to isolate the membrane. In order to form yolk-shell-structured nanoparticles, they first coated liposomes with a lipid bilayer coating (LM), then wrapped them with MCF-7 cell membrane (CCM) or 1,2-dimyristoyl-sn-glycero-3-phosphocholine (DMPC), respectively, to form CCM@LM and L@LM, respectively, using mesoporous silica nanoparticles (MSN).	Mesoporous silica nanoparticle	PEGylated liposome	Doxorubicin and mefuparib hydrochloride	Cancer chemotherapy	Targeted drug delivery. NPs coated with CCM and with a yolk-shell structure were evaluated for cancer chemotherapy. In addition to its homologous tumor-targeting ability due to the CCM coating, the resulting formulation (CCM@LM) exhibited a favorable immune escape profile. The limitation of this study is that we have to be careful while isolating the membrane from cancer cells regarding any residual cells’ presence.	Significantly improved the antitumor effect compared to chemotherapeutic drugs (Doxil)	[27]
6.	Erythrocytes. RBC membrane RBCM was formed by breaking up RBCs extracted from nude mice, and then incubating them under low osmotic pressure. As RBCM is sonicated, its size degrades from micro to nano. Perfluorocarbon (PFC) nanoparticles were encapsulated within biocompatible poly(d,l-lactide-co-glycolide), PLGA, resulting in PFC@PLGA nanoparticles, which were then coated with RBCM.	Perfluorocarbon nanoparticles	PLGA	-	Cancer radiotherapy	Therapy. By diffusing oxygen through blood vessels, PFC@PLGARBCM with nanoscale sizes could improve the overall oxygenation status of the tumor after i.v. injection and the tumor is relieved from hypoxia, which can enhance the tumor inhibition by radiotherapy (RT). The limitations exist regarding the oxygen supply to the interior part of the tumors, which may inhibit the necrosis of the tumor.	Delivery of oxygen and favorable for cancer treatment	[28]
7.	Platelet. A freeze-thaw process was used to extract platelet membranes. A model drug for rheumatoid arthritis (RA), FK506-loaded nanoparticle cores were prepared by the process of nanoprecipitation. Platelet-mimetic nanoparticles (PNPs) were prepared by mixing PLGA nanoparticles with platelet membrane solutions and sonicating them to fuse the membrane onto the cores of the nanoparticle.	PLGA nanoparticles	-	Model drug- FK506	Rheumatoid arthritis	Targeted drug delivery. CIA mouse model of RA showed significant RA progression control with FK506-PNPs, and preliminary safety studies showed excellent biocompatibility for PNPs. Limitations include the scaling up of the ghost cells, i.e., the platelet membrane requires human platelets, which has ethical concerns.	Accurate accumulation of formulation in the inflammatory synovial tissue.	[29]
8.	Cancer cell. PLGA nanoparticles, containing siRNA and dox was prepared by water in oil emulsion method. Hela human cervix carcinoma cells and MDA-MB-231 human breast cancer cells were suspended in typical hypotonic lysing buffer and lysed in ice bath with repeated freezing and thawing. The membranes were collected using repeated centrifugation. In order to obtain membrane vesicles, the above cancer cell membrane fragments were extruded for 20 passes through a 400 nm polycarbonate membrane. To coat the membrane vesicles onto PLGA cores, nanocores and membrane vesicles were co-extruded through a 200 nm polycarbonate membrane.	PLGA nanoparticles	-	Doxorubicin and PD-L1 siRNA	Cancer therapy	Targeted drug delivery. PLGA nanocores loaded with doxorubicin (Dox) and siRNA targeting PD-L1 (si.PD-L1) were constructed, camouflaged, and functionally modified using a cancer cell membrane (CCM). In addition for targeting homologous source cells, CCMNPs also have great potential as a platform for guiding the delivery of homologous-targeting therapeutics. PLGA nanoparticles cloaked in Hela membranes exhibited more powerful cellular internalization when compared with bare PLGA nanoparticles, while MDA-MB-231 cells showed reduced nanoparticle binding. Cell membrane isolation from cancer cells may also contain some unlysed cells which may contaminate the product, making this a limitation of this study. Moreover, the extracellular matrix of cancer cells may impart deleterious effects on normal cells which needs to be addressed.	Selective accumulation and sustained delivery of drugs	[30]
9.	Macrophage. Solvothermal method was used to synthesize Fe_3_ O_4_ NPs. Membrane-derived vesicles (MM-vesicles) were prepared using RAW 264.7 cells that were suspended in hypotonic lysing buffer containing EDTA-free mini protease inhibitor tablet. The cells were then subjected to Dounce homogenizer for disruption. After isolating the membranes using centrifugation, the MM-vesicles were extracted by physical extrusion of the pellets. The pellets were passed several times through 400 nm and 200 nm microporous membranes using an Avanti mini extruder. Fe_3_O_4_ NPs synthesized earlier were mixed with MM-vesicles and extruded through a 200 nm membrane 11 times and the additional MM-vesicles were removed using an external magnetic field; the resultant Fe_3_O_4_@MM NPs solution was left in PBS.	Magnetic iron oxide	-	-	Breast cancer therapy	Photothermal therapy. MM-vesicles (macrophage membrane-derived vesicles) were collected from macrophages and then coated on Fe_3_O_4_ NPs. A macrophage membrane camouflaged nanoparticle (Fe_3_O_4_@MM NPs) inherited good biocompatibility and immune evasion properties and was capable of targeting cancer and converting light to heat. It could be used for enhanced photothermal tumor therapy. The fascinating properties of macrophage membrane coatings in evading immune cells and targeting cancer require further investigation. Limitations include the scaling up of membranes from macrophages.	Exhibited great biocompatibility and light-to-heat conversion capabilities	[31]
10.	Erythrocytes. The Prussian blue nanoparticles (PB NPs) were prepared using the precipitation method using citric acid as a capping agent. The whole blood was collected from the eyeball of female KM mice and centrifuged for plasma removal. The RBCs were hemolyzed using distilled water and the membrane was selected using centrifugation. The vesicles were collected by sonication of the membrane followed by a series of extrusions using 400 nm and 200 nm polycarbonate membranes. Ce6 solution was added to these vesicles for binding and excess Ce6 was removed by centrifugation. To prepare PB@RBC/Ce6 NPs, PB NPs were added to RBC/Ce6 vesicles prepared previously and extruded using 100 nm membrane several times to yield the final product, PB@RBC/Ce6 NPs.	Prussian blue nanoparticles		Chlorin e6	Dual cancer therapy	Photothermal and photodynamic therapies. Prussian blue nanoparticles (PB NPs) coated with photosensitizing agent Chlorin e6 (Ce6)-embedded RBC membrane vesicles, named PB@RBC/Ce6 NPs, were synthesized. A nude mouse orthotopic tumor model was used to assess the cytotoxicity and therapeutic efficacy of PB@RBC/Ce6 NPs in vivo and in vitro assay was done using 4T1 cell line. The findings of the study suggested that erythrocyte membranes are efficient carriers of the photosensitizer Ce6 due to hydrophobic interaction. They could impart efficient PTT with higher biocompatibility and higher endocytosis in tumor sites imparted synergistic PDT and PTT-mediated cell killing to inhibit cancerous tumor growth. Limitations of this study may be the ethical considerations in the scaling up of the erythrocyte membranes.	Produced a notable effect in boosting the necrosis and showed a synergistic therapeutic effect	[32]

### 3.5. Bacterial Cell Nanodecoys (BCNDs)

The nanosized outer membrane vesicle (OMV) of a bacterium holds great potential in drug carrier applications. The OMV will be generated by both Gram-positive and Gram-negative, pathogenic, and non-pathogenic bacteria. This vesicle contains necessary materials which contribute to the maintenance of the ecosystem of bacteria. Proteins, nucleic acids, lipids, and virulence factors are the major constituents of OMVs. Studies revealed that OMVs encapsulated with nanoparticles can actively transport the cargo to perform desired functions. OMVs are capable of especially controlling the counteractions of target organisms and host responses, and so OMVs hold a remarkable position in biomedical applications. Hence, further coating of OMVs with nanoparticles can fetch great advantages. Gao et al. fabricated gold nanoparticles coated with OMVs (OMV-AuNP). The size of the obtained particle was measured as 30 nm in diameter. The in vivo studies on vaccinated mice showed rapid maturation of dendritic cells of lymph nodes. Compared to OMVs, vaccination with OMV-AuNP showed high avidity and generated a strong CD4^+^ biased cellular response towards the target pathogenic bacteria [33]. Chen et al. synthesized polymeric micelles coated with OMVs. The drug tegafur was loaded into the micelles. The ability of the particle to inhibit the metastasis was evaluated using a metastasis inhibition assay. The in vivo study results showed higher efficiency in the inhibition of metastasis, specifically in lung cancer, and exhibited cancer immunotherapeutic properties [34]. Silica nanoparticles are one of the widely utilized nanoparticles for drug-carrying applications due to their porous structure. Wu et al. developed a biomimetic system to enhance the targeting and uptake of the antibiotic drug rifampicin. Mesoporous silica nanoparticles were prepared and loaded with rifampicin, which was then encapsulated on the OMVs, and extracted from *E.coli.* The in vivo and in vitro results demonstrated that the prepared camouflage particle exhibited good biocompatibility and improved antibacterial effect against Gram-negative infections, and completely eradicated the target bacteria compared to free rifampicin [35].

### 3.6. Cancer Cell Nanodecoys (CCNDs)

Coating nanoparticles with cancer cell membranes can facilitate the delivery of the payload to attain high-precision tumor therapy due to their homologous binding capabilities. Rao et al. worked on cell membrane-coated nanoparticles for personalized cancer therapy. The group used a cancer cell membrane derived from a xenografts model for the coating onto cisplatin-loaded gelatin nanoparticles. The resulting construct was tested on the subcutaneous model and post-surgery model to check the tumor ablation and recurrence, respectively. The biomimetic material showed complete ablation of the tumor and inhibited the recurrence. Hence, the above material was represented as an effective strategy for cancer treatment [36]. Zhao et al. developed cancer cell membrane-coated mesoporous silica nanoparticles. The particle was tagged with an antibody (anti-programmed cell death protein 1) and loaded with dacarbazine. In comparison to free dacarbazine, the in vivo and in vitro results of nanoformulated dacarbazine demonstrated improved tumor targeting and activated tumor-specific T cells. The formulation regulated the immunosuppressive tumor microenvironment and suppressed melanoma growth altogether [37]. Jin et al. recently reported the chemo-photodynamic application of cancer cell membrane-coated upconversion nanoparticles (UCNPs). The particle also consisted of rose bengal as a photosensitizer, doxorubicin as a drug, polyethylene glycol-thioketal as ROS sensitive polymer, and anti-CD73 antibodies as a targeting agent. The particle displayed the synergistic effect of chemo-photodynamic therapy and immunotherapy by selective tumor killing and escaping the immune responses. The UCNPs here worked as near-infrared (NIR) sensors to shift the NIR to visible light upon irradiation after administration. The visible light thus formed the ROS due to the presence of a photosensitizer, which would then facilitate the rupture of the vesicle and drug release. The antibodies specifically reached the tumor microenvironment, thus contributing to selective tumor killing. Hence, this work could be a potential strategy for metastatic cancer treatment [38].

### 3.7. Hybrid Cell Nanodecoys (HCNDs)

Coating nanoparticles with biologically derived membranes provides excellent advantages. In comparison to a single membrane-coated nanoparticle, a hybrid membrane offers numerous advantages since it displays the characteristic of both the source cells. Hybrid nanodecoys are applied mostly in tumor applications and vaccinations. Researchers that developed HCNDs have reported that the HCNDs escaped the immune system well, featured long circulation time, and specifically targeted the tumor cells due to the membrane proteins. Recent research findings on HCNDs have been tabulated in Table 2.

## 4. Applications

**Figure 2:**
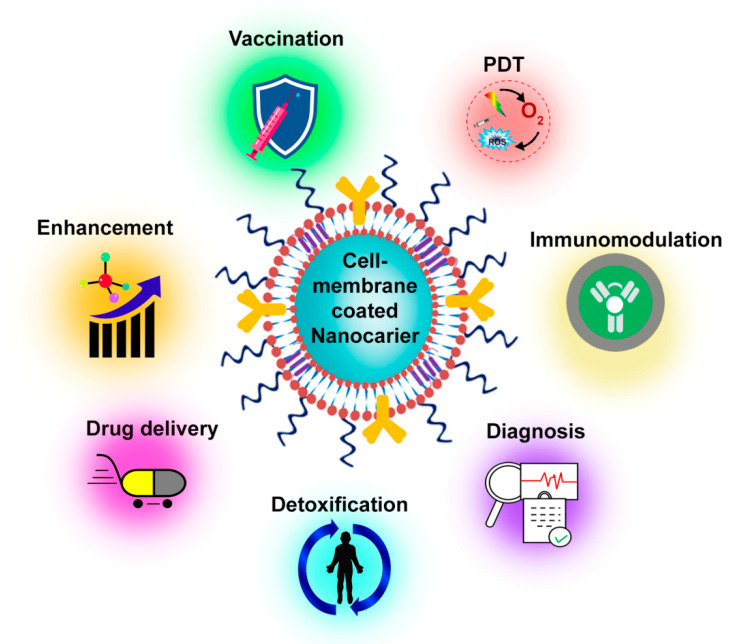
Biomedical applications of nanodecoys.

The bio-mimicking ability of the nanodecoys allows them to function inside the biological system with negligible adverse effects. Thus, they are considered the best candidate for therapeutic applications, namely, drug delivery, detoxification, vaccination, immunomodulation, photodynamic therapy, etc. (Figure 2). Owing to their multifunctional properties, they are also being used to carry diagnostic agents for bioimaging applications. Integration of diagnosis and therapeutic characteristics of nanodecoys lead to theranostic applications where a single particle could perform both activities simultaneously.

### 4.1. Bioimaging

Anomalies of the internal structures, the presence of foreign bodies, and the state of disease condition have been examined to design appropriate therapeutic procedures. As for cancer, there are two components of detection: (i) Screening and (ii) Downstaging, i.e., early diagnosis. Screening refers to testing a healthy individual for the presence of a tumor even before the symptoms, whereas early detection is testing over the appearance of minor symptoms. Early detection of cancer provides a high curable rate with long-term management. For improved information on anatomical and functional activities of the disease condition, nanostructures are being employed, mostly in oncology. Due to the ease of tuning the physical and optical properties, nanoparticles are excellent candidates in the imaging field. Surface functionalized nanoparticles can indisputably distinguish between healthy tissues and abnormal lesions. Present contrast agents (CA) exhibit fast metabolism, and tumor tissue detection is limited due to the spatial resolution that is generated by the hardware of imaging modalities. The distribution is usually non-specific, which in turn affects the resolution of images. Therefore, the CA can be effectively transported to the tumor site through nanocarriers. There are four ways where nanoparticles can help in the imaging field: (i) NPs as CA: optical properties of the nanoparticles such as upconversion nanoparticles (UCNPs) aids in imaging applications, (ii) NPs as a carrier of CA: to deliver CA with other imaging elements, nanoscaled carrier systems are being used, (iii) NPs to detect biomarkers: detection of biomarkers in certain cancer types are very arduous, while studies found that nano-based biosensors can significantly amplify the signal from tumor area, (iv) NPs to spot circulating tumor cells (CTCs): NPs are found to be a standalone material as a CTC capture device. Rao et al. demonstrated that erythrocyte membrane-coated UCNPs surface functionalized with folic acid receptors for specific binding. The in vivo study showed enhanced tumor imaging with negligible systemic toxicity [51]. For highly specific tumor imaging, the same group synthesized UCNPs coated with a cancer cell membrane that was sized about 80 nm. The surface of the particle was further modified with PEGylated phospholipids, which were sized about 10 nm. Both the cancer cell membrane and UCNPS showed fluorescence emission. The same formulation coated with erythrocyte was employed as control. The in vivo investigation was carried out in a mouse model. The overall results suggested that the CCNDs can be remarkably used in highly specific tumor imaging. To test the virulence factor of the membrane derived from the cancer cell, it was injected into an immunodeficient mouse and showed no tumor development [52]. Zhang et al. employed cancer cell membranes to encapsulate rare-earth doped nanoparticles (REDNPs). For comparison, REDNPs were encapsulated with PEG and a similar treatment was provided. Compared to the PEGylated particle, CCNDs showed enhanced imaging of tumor cells in NIR-II window with decreased relative uptake by the spleen and liver [53].

### 4.2. Drug Delivery

Site-specific delivery can be achieved by nanoscaled materials. Transportation of drugs specifically to the diseased site could significantly minimize the toxic effects. Recently, the naturally derived membrane coating of nanoparticles has gained more attention since it enhances the properties of a particle. There are three major highlights of membrane coating: (i) due to the presence of surface protein on cell membranes, coating them with nanoparticles can improve the targeting and attain selective accumulation on diseased sites, especially in the tumor microenvironment, (ii) due to the biomimetic membrane characteristics, it can readily escape the biological barriers and immune responses, (iii) the circulation time of the particle in the bloodstream can be prolonged. Overall, nanodecoys have emerged as a promising class of theranostic agents in managing and treating a plethora of diseases [7]. The research team of Chen recently utilized an erythrocyte membrane to formulate a nano-based drug carrier vehicle for delivering paclitaxel. A lipid insertion method was used to prepare the vehicle tagged with bispecific recombinant protein for specific tumor targeting. The formulation was tested for stability and showed in vitro stability for 8 days. The prepared particle was sized about 171 nm and was spheroid in shape. The release profile was studied, which showed a biphasic pattern. The in vivo results conducted on the gastric cancer-bearing mouse model demonstrated a specific accumulation of drugs on the target site and eliminated about 61% of tumor volume [54]. Li et al. fabricated a drug delivery vehicle by coating macrophage-derived microvesicles onto PLGA nanoparticles loaded with tacrolimus as a model drug. The particle was tested on an in vivo mouse model induced with rheumatoid arthritis. The team used a novel method to prepare the macrophage by implying cytochalasin B and analyzed it through iTRAQ technology (isobaric tags for relative and absolute quantitation). The particle was further tagged with two different dyes: DiR and DiD to obtain multicolor imaging. For the comparison, bare nanoparticle and erythrocyte-coated nanodecoys were used. After the intravenous administration of three sets of samples and control to the mouse, the evaluation showed increased efficiency in macrophage-coated NPs [55]. Thrombocytes, being the smallest blood cells, exhibit superior functionalities as they can escape phagocytosis by macrophages. Hence, it is believed that TNDs can prolong circulation in vivo. Wang et al. developed a drug carrier by encapsulating PLGA nanoparticles in a platelet membrane shell to deliver bufalin. The in vivo and in vitro studies revealed that the prepared particle showed a sustained drug release profile and targeted release of the drug. The toxicity of the formulation was evaluated in a cancer-induced mouse model to analyze the biosafety and tumor inhibition rate. The overall results demonstrated that the TNDs performed site-specific drug delivery with minimal adverse effects [56].

### 4.3. Photodynamic Therapy (PDT)

Studies have demonstrated that PDT is an effective, clinically approved therapeutic procedure for treating cancer and pre-cancer due to its minimum invasiveness and minimal cytotoxic activity. The principle of PDT depends on the generation of ROS by the photosensitizer that destroys cells [57]. After administration of the dye, the site that needs to be treated alone can be irradiated to avoid killing healthy cells. Since this occurs only when it is irradiated, it holds several advantages. Encapsulation of photosensitizer in a nanoparticle can reach the site precisely and perform selective accumulation on tumor cells, which minimizes the distribution level and increases the bioavailability of the given photosensitizer. Still, PDT is not being chosen as the first-line treatment of cancer due to certain shortcomings, and it is one of the most preferred procedures during a situation where the condition does not allow surgical removal. Knowledge about the fate of nanoparticles after performing the programmed functions is still lacking; furthermore, the cytotoxic profile of nanoparticles needs a forum to discuss in detail before employing them in real-time applications such as PDT to prevent other infections. Unlike radiation-mediated treatment procedures, PDT can be repeated several times and offer long-term cancer management where a complete cure is impossible. To overcome the limitations that nano-mediated PDT currently faces, cell membrane-coated nanoparticles, so-called nanodecoys, can be employed. The biocompatibility of nanoparticles can be enormously enhanced by coating them with nanodecoys. For the core to encapsulate photosensitizers, mesoporous silica nanoparticles (MSN) are being widely used due to their porous structure and easy surface modification. Wang et al. recently worked with HCNDs, which were prepared with MSN as a core to encapsulate hypoxic prodrug, tirapazamine, and a photosensitizer, indocyanine green, coated with erythrocyte membrane and cancer cell membrane. Since the hypoxic condition of the tumor microenvironment is one of the limitations of PDT, the team co-loaded the hypoxic prodrug along with photosensitizer. The in vivo experimental results performed on a tumor-bearing mice model showed tumor inhibition rates of 34% and 64% for the MSN encapsulated co-drug loaded nanoformulation and membrane-encapsulated nanoformulation, respectively [58]. Peng et al. extracted OMVs from *E.coli* bacterium. The nanoscaled membrane was further modified with indocyanine green and targeting ligand. Upon irradiation to near-infrared light, the particle synthesized featured photodynamic activities along with photothermal and apoptosis induction by TRAIL (tumor necrosis factor-related apoptosis-inducing ligand). This showed enhanced therapeutic performance and complete eradication of skin melanoma [59]. Zhao et al. constructed a metal–organic framework (MOF) (FeTCPP/Fe_2_O_3_) by incorporating ferric oxide and a porphyrin-based photosensitizer via the liquid diffusion method. In order to enhance biocompatibility and circulation time, the formulation was coated with an erythrocyte membrane and tagged with synthetic DNA aptamer (AS1411). After excellent results were obtained from the in vitro study conducted with human epithelial carcinoma cells, the study was further carried out on an in vivo mouse model. The MOF nanomaterial showed improved PDT effects in ENDs [60].

### 4.4. Theranostics

Integration of the diagnostic and therapeutic abilities of a single particle will enable it to perform both functions simultaneously. These multifunctional nanoparticles possess several advantages, including enhanced imaging of tumor sites, site-specific drug delivery, enhanced EPR (enhanced permeation and retention effect), and monitoring [61,62,63,64,65,66]. Thus, theranostic particles are highly beneficial for planning personalized medicine (Table 3). Encapsulation of theranostic particles onto cell membranes can further improve biocompatibility by eliminating possible interactions with the immune system. There are four general ways to fabricate a theranostic particle: (i) therapeutic agent can be encapsulated or conjugated onto imaging NPs such as SPION, UCNPs, quantum dots, etc. [67,68,69], (ii) CA can be tagged onto a therapeutic nanoparticle such as silver nanoparticles, etc. [70], (iii) both the CA and therapeutic agent can be encapsulated onto a biocompatible NPs such as polymeric NPs, nano-vesicles, MSN, etc., (iv) engineering of unique NPs with imaging and therapeutic potential is possible with PEGylation to improve biocompatibility. Despite continuous efforts, theranostic NPs are still in translational stages due to three major concerns: insufficient data on the systemic evaluation of biosafety of the particle, cytotoxicity, and obtaining expected clinical effects. Rao et al. extracted membranes from erythrocytes to encapsulate magnetic NPs via a microfluidic electroporation method with a size of about 60 nm in diameter. The properties of both the membrane coating and the core were blended to produce image-guided therapy. Magnetic NPs in the core exhibited excellent magnetic and photothermal properties. The membrane coating could improve the biocompatibility and circulation time of the particle in the bloodstream. Thus, the prepared nanostructure was used to perform the magnetic resonance imaging (MRI) assisted photothermal therapy when tested in vivo [71]. Recently, Li et al. reported dual model image-guided photodynamic therapy for tumor treatment. For the core material, SPION crosslinked with styrene and acrylic acid and sized about 9 nm was used. The surface of the particle was then coated with polyethyleneimine (PEI) through electrostatic interaction, and the chlorin e6 photosensitizer was tagged along with PEI. Two sets of particles were prepared for comparison: core-encapsulated photosensitizer and core-encapsulated photosensitizer-coated tumor cell membrane. Both sets were tested for the ability to generate ROS and the MR/NIR fluorescence imaging on an in vivo mouse model. The results demonstrated that CCNDs provided better efficiency than bare NPs [72]. Gene expression is one of the major causative conditions for cancer, and the overexpression of certain genes is noted in most of the tumors. Planning gene therapy along with conventional treatment techniques is considered one of the best beneficial treatment strategies. Mu and colleagues designed stem cell membrane-coated magnetic nanoparticles to deliver siRNA. The siRNA could effectively inhibit the expression of the polo-like kinase-1 (Plk-1) gene, which would further cause apoptosis. The synthesized particle provided image-guided combinational photothermal and gene therapy when tested on an in vivo mouse model bearing prostate cancer [73]. The different types of nanodecoys used in theranostics are given in Table 3.

**Figure 3:**
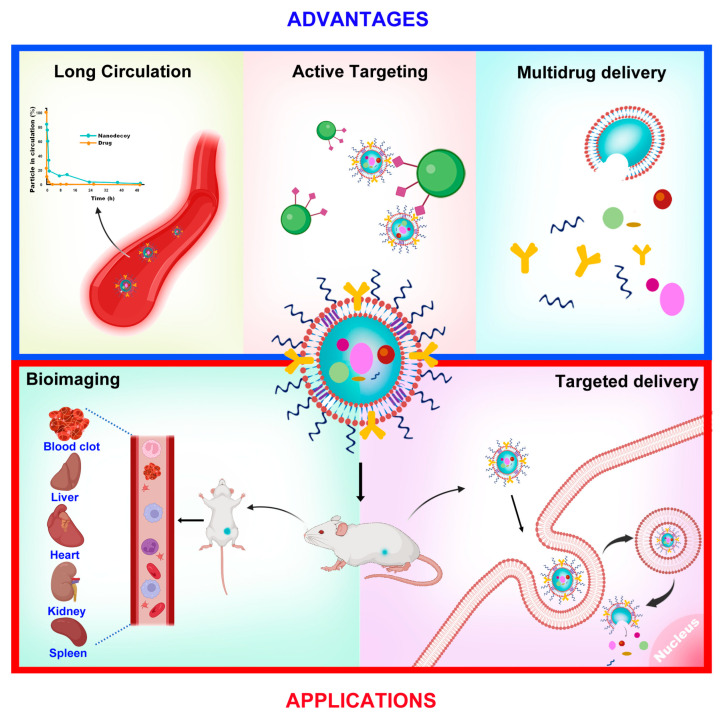
The advantages and theranostic applications of nanodecoys.

The advantages and applications of nanodecoys are depicted in Figure 3.

### 4.5. Other Applications

The above-mentioned applications are some of the renowned fields where nanodecoys are widely used. Other applications include immunomodulation, detoxification, enhancement of particle properties, and vaccination. The outer surface of nanodecoys consists of several sites for binding with complementary-shaped receptors or antibodies. Several researchers are currently involved in developing nanodecoys for immunomodulation [78]. The toxicity of the particle, i.e., the core properties, could be effectively controlled by a biocompatible coating of the cell membrane; thus, they could enhance the characteristics. Toxins are secreted by various organisms for their survival, which could bind to a nearby cell, deform the shape, and disrupt normal metabolic functions. Hence, nanodecoys during this stage could be employed to detoxify the toxin moieties. Polymeric nanomaterials and nanosponges are two widely used materials for detoxification applications. Recently, Gong et al. reported the synthesis of polymeric nanoparticles encapsulated in the mitochondrial cell membrane. The outer mitochondrial membrane (OMM) was capable of binding with B-cell lymphoma protein inhibitor molecule (ABT-263) and protected cells from ABT-263 mediated apoptosis [79]. Chen et al. demonstrated the neutralization behavior of erythrocyte membrane-coated polymeric nanosponges. The prepared formulation acted as a toxin decoy and effectively neutralized the broad spectrum of hemolytic toxins [24]. Cell membranes are also being produced, modifying the general features for them to perform better. Owing to this, Park et al. engineered a genetically modified cell membrane to encapsulate polymeric nanoparticles loaded with dexamethasone to treat inflamed lungs. The inflamed lungs overexpress VCAM-1 (vascular cell adhesion molecule-1. The cell is genetically modified to express VLA-4 (very late antigen-4), which can specifically target and bind to the VCAM-1 of inflamed lungs, thus providing targeted drug delivery. The results obtained from the work suggest that the cell membranes can be tailored in a desired way to perform particular functions [80]. Another important field of application of nanodecoys is vaccination. The development of vaccination to provide cancer immunotherapy is of great interest currently. Nanodecoys that have been engineered for cancer immunotherapy are tabulated in Table 4.

## 5. Considerations in Applications of Nanodecoys

Despite several advantages of nanodecoys that are still in preliminary research, there exist certain limitations. The very first stage of preparation itself is crucial, since most of the integrity and homogeneity are lost in isolation procedures. Hence, the process chosen for the isolation and coating of nanoparticles plays a vital role in the efficacy of the formulation. For cancer treatment, before encapsulating the drugs, the stability of the formulation should be determined to avoid the leakage of a drug in inappropriate sites, since stability is crucial to control durability. Usually, in the bloodstream or tumor microenvironment, the natural cells deform and lose their functions. This is due to the loss of rigidity of the cell membrane caused by intercellular cytoskeleton disruption. This is the reason the stability of the particle in different surroundings needs to be crosschecked before experiments. A recent investigation by Liu et al. addressed one of the effects of integrity on the final formulation. The researchers performed fluorescence quenching assay, in vitro homologous targeting studies, and molecular simulations combined with experimental analysis to monitor the integrity of the cell membrane. The quantitative method developed in this study excellently validated the integrity, and other fundamental understandings about nanodecoys are also documented. In conclusion, it was found that current coating procedures fail to provide integrity and are only partially coated. The partially coated particles were studied for the internalization into target cells using in vitro homologous targeting studies. The degree of coating of the cell membrane on nanoparticles decided the stability, where highly coated particles entered the cell individually, and particles with a low degree of coating aggregate before the entry and entered via cooperation mechanism. Thus, it is clear that the determination of the ratio of coating needs to be studied to achieve successful synthesis and desired application [88]. Another important concern regarding nanodecoys is the insufficient data on regulatory guidelines for their usage. Nanodecoys need a forum to discuss and draw conclusions about regulatory guidelines and instructions. Hence, concerns about nanodecoys should be sorted out to employ them in future biomedical applications.

## 6. Conclusions

Nanodecoys have significantly contributed to revolutionizing the nanotherapeutic industry. They possess the abilities of both the drug enclosed as well as the cell, which allows them to present optimum results. This enhances the capabilities of nanodrugs to another level and represents a substantial developmental step in drug targeting systems. The therapeutic capability of nanodecoys has gained immense attention in biomedical research. The limitation mentioned, if solved, could provide excellent therapeutic efficiency. Before employing it in real-time application, the best method of isolation can be determined to greatly control the integrity of nanodecoys. Experiments that can test the stability, structural characteristics, and responsiveness to the physiological ambiance need to be performed to gain enough knowledge about the particle. In this manner, the concerns regarding nanodecoys can be tackled. Working with different membranes and nanoparticles can help in the treatment of a plethora of diseases, such as cancer, atherosclerosis, diabetes, pulmonary diseases, and many more.

## Figures and Tables

**Table 2:** Recent Advances in Hybrid Nanodecoys for Biomedical Applications.

S. No	Cell Membrane/Extraction and Coating Method	Nanoparticles	Surface Modifications	Drugs	Target Cell/Disease/Pathogen	Applications/Functions and Limitations	Key Features	References
1.	Neutrophil/Erythrocyte. From C57 mice, erythrocyte membrane was collected by lysing the RBCs and repeatedly centrifuging them to remove the hemoglobin. Neutrophil was obtained from the mouse leg (six weeks old, male, C57) bone marrow through density gradient centrifugation. The neutrophil was added to membrane protein extract that contained protease inhibitor for cell lysis, and further homogenized and centrifuged to obtain the neutrophil membrane. Cell membrane-coated nanoparticles were engineered by taking both the membranes, erythrocyte, and neutrophils, followed by sonication. Further, the CuS NPs or Dexp-loaded CuS NPs (D-CuSNPs) were added into the mixed membranes and sonicated. The final product was isolated using centrifugation.	Copper sulfide nanoparticles	-	-	Osteoarthritis	Anti-inflammatory photothermal therapy. Fusion membrane nanocomposite was made from the neutrophil membrane and erythrocyte membrane and was coated on the surface of dexamethasone sodium phosphate (Dexp)-loaded CuS NPs (D-CuS@NR NPs). D-CuS@NR nanoparticles possess excellent photothermal conversion capacity, controlled drug release behavior, and good cytocompatibility in vitro. In comparison with free Dexp and D-CuS@NRNPs, D-CuS@NRNPs combined with 1064 nm NIR therapy showed a greater antiinflammation effect at the cellular level. D-CuS@NR NPs possessing CD11a activate the neutrophil membrane in inflammatory joints and can target OA sites as shown in vivo fluorescence imaging. The limitation of this research is that the fused membrane is prepared from cells of human origin, which can have ethical concerns and scaling-up issues.	Enhanced cytocompatibility, anti-inflammatory ability, and upon irradiation with NIR, showed photothermal responsive drug release	[39]
2.	Dendritic cells/Cancer cells. A polymeric (PLGA) nanoparticle core was fused with multiple antigens derived from tumor cells, resulting in cancer cell membrane-coated nanoparticles (CCNPs), which facilitate cell internalization. After that, bone marrow-derived DCs (BMDCs) were incubated with CCNPs for antigen processing and presentation. They generated DC membrane-coated nanoparticles by extracting membranes containing tumor-associated antigen epitopes from CCNPs-pulsed BMDCs and coating them onto polymeric nanoparticle substrates.	-	-	-	T-Cells	Immunotherapy. The antigens on cancer cell membranes are extracted and coated on a nanoparticle substrate that can be readily ingested by dendritic cells (DCs) for antigen presentation and processing. Biomimetic nanoparticles (BNs) are formed by fusing DC membranes presenting related antigen epitopes to a nanoparticulate core. A number of tumor models were shown to benefit from BNs, including ovalbumin-expressing B16 (B16-OVA), HPV E6 and E7-expressing TC-1, and Hepa 1–6 tumor-bearing mice. The limitation of this research is that the fused membrane is prepared from cells of human origin, which can have ethical concerns and scaling-up issues. Moreover, cancer cell membranes have the capability of remodeling the normal cell ECM.	Exhibited desirable tumor regression and survival rate.	[40]
3.	Platelets/Leukocytes. Platelets(PLTs) and leukocytes (WBCs) from human blood was collected by centrifugation and the membranes were isolated after removing their intracellular contents using a combination of hypotonic lysis, followed by mechanical disruption and gradient centrifugation. The platelet membrane (PM) and leucocyte membranes (WM) were extruded through a mini extruder repeatedly to form vesicles of hybrid membranes (HMs). Then, magnetic beads (MBs) and HMs were mixed by sonication and extrusion. The final products, PLT–WBC hybrid membrane-coated immunomagnetic beads (HM-IMBs), were synthesized by step by- step conjugation of the surface of HM-MBs with 1,2-distearoylsn- lycerol-3-phosphoethanolamine-N-[methoxy(polyethylene glycol)-2000]-COOH (DSPE-PEG-COOH), streptavidin (SA), and biotinylated anti-EpCAM.	Immunomagnetic beads	Anti-EPCAM antibodies	-	Breast cancer	Theranostics. A platelet-leukocyte hybrid membrane (HM) is formed by fusing platelet membrane (PM) and leucocyte membrane (WM), coating it on magnetic beads (MBs), and then modifying its surface with CTC-targeting antibodies. It is possible to isolate circulating tumor cells (CTCs) highly efficiently and highly specifically using the hybrid membrane-coated immunomagnetic beads (HMIMBs), which have enhanced cancer cell binding ability from PLTs and reduced homologous WBC interaction from WBCs. The limitation of this research is that the fused membrane is prepared from cells of human origin, which can have ethical concerns and scaling-up issues.	Specifically, isolate circulating tumor cells and detect PIK3CA gene mutations	[41]
4.	Erythrocytes/Cancer cells. RBC-M hybrid membrane-coated melanin (Melanin@RBC-M) nanoparticles were synthesized by extrusion method by mixing the two membranes (RBC membrane and MCF-7 cell membrane vesicles) at different ratios to the melanin nanoparticles, followed by a series of extrusions using polycarbonate porous membrane and sonication. The excess membranes were excluded by centrifugation and the final product was retained.	Melanin nanoparticles	-	-	Breast cancer	Photothermal therapy. An erythrocyte-cancer (RBC-M) hybrid membrane was made by fusing RBC membrane with MCF-7 cell (human breast cancer cell line) membrane. The fabricated membrane camouflaged the melanin nanoparticles (Melanin@RBC-M) for photothermal therapy executed in vivo. Melanin@RBC-M prepared with 1:1 membrane protein weight ratio of RBC membrane to MCF-7 membrane showed enhanced PTT efficacy in comparison to other Melanin@RBC-M made with other membrane ratios, as well as pristine melanin nanoparticles. The reason was attributed to the optimal balance between homotypic targeting and prolonged circulation in the blood. The limitation of this research is that the fused membrane is prepared from RBCs of human origin, which can have ethical concerns and scaling-up issues.	Improved the photoacoustic signal to perform photothermal anticancer therapy	[42]
5.	Cancer cells/Erythrocytes. Fe_3_O_4_ nanoparticles were prepared according to the solvothermal method using FeSO_4_·7H_2_O as precursor. Fe_3_O_4_-ICG nanoparticles were prepared by immobilizing negatively charged ICG onto positively charged Fe_3_O_4_ nanoparticles by virtue of electrostatic interactions. To extract cell membranes from ID8 cells, the harvested cells were added to a hypotonic lysing buffer which contained membrane protein extraction reagent and protease inhibitor of phenylmethanesulfonyl fluoride (PMSF). Then, the lysate was sonicated and centrifuged to obtain IG8-M. For RBC-M, whole blood was obtained from orbital sinus of C57BL/6 mice, centrifuged to remove plasma, WBCs, and platelets, and washed with PBS. After subjecting the RBC pellet to a hypotonic solution, the lysis was performed, hemoglobin was removed by repeated centrifugation, and RBC-M was obtained. The RBC-M was added to DiO/DiIdouble dye-labeled ID8-M at the different membrane protein weight ratios, followed by sonication to obtain IRM. By the ultrasonic method, the final product Fe_3_O_4_-ICG@IRM nanoparticles were obtained by mixing Fe_3_O_4_-ICG solution with the IRM solution followed by sonication at ice.	Magnetic Nanoparticles	-	Indocyanine green	Ovarian Cancer	Photothermal/Immunotherapy. In order to achieve synergistic photothermal-immunotherapy of ovarian cancer, the researchers constructed a hybrid membrane consisting of ID8 cell membranes (ID8-M) and RBC membranes (RBC-M) and coated them onto ICG-loaded magnetic nanoparticles (Fe_3_O_4_-ICG@IRM). In vivo, Fe_3_O_4_-ICG@IRM demonstrated outstanding performance in photothermal immunotherapy for ovarian cancer due to the combination of the core nanoparticles (Fe_3_O_4_-ICG) with their inherent photothermal conversion properties, membrane antigens, and the resulting whole-cell tumor antigens. The limitation of this research is that the fused membrane is prepared from erythrocytes, which can have ethical concerns and scaling-up issues.	Prolonged the circulation lifetime in the bloodstream and possessed specific recognition of target cells in vitro and in vivo	[43]
6.	Bacterial Vesicle/Cancer Cells. Hollow polydopamine (HPDA) nanoparticles were synthesized by hydrothermal method using dopamine and SiO_2_ nanoparticles. The SiO_2_ nanoparticles were synthesized initially by precipitation method using TEOS as precursor and used for HPDA nanoparticle synthesis. For preparing cancer cell (CC) membrane, B16-F10 cells were harvested using cell scraper, collected by centrifugation, and added to the membrane protein exaction kit containing PMSF. Further cell lysis was done by freezing and thawing, and centrifugation was used to collect the CC membrane. E. coli DH5α cell outer membrane vesicle (OMV) was obtained by subjecting the cell pellet to OMVs’ Amicon centrifugal filters. To obtain a hybrid OMV-CC membrane, sonication was employed to a mixture of CC membrane and OMV at a particular ratio. For wrapping the hybrid membrane over HPDA NPs, the OMV-CC membrane mixture was mixed with HPDA nanoparticles and treated ultrasonically, followed by centrifugation to remove the unbound membranes.	Polydopamine nanoparticles	-	-	Melanoma	Tumor-Specific Immune Activation and Photothermal Therapy. In order to enhance the efficacy of cancer therapy, they synthesized a hybrid cell/OMV (outer membrane vesicle) membrane with multifunctionalities and used it to coat nanoparticles. The OMV was derived from E. coli DH5α and cancer cell (CC) membrane (B16-F10 cell membrane) and was fused, resulting in an OMV-CC hybrid membrane. It was encapsulated inside hollow polydopamine (HPDA) NPs to obtain the final nanoparticle, namely, HPDA@[OMV-CC] NPs. OMV immunotherapy, along with HPDA-mediated photothermal therapy, could completely eradicate the melanoma without any significant adverse effects. Limitations of this study may be the fusion of bacterial cells with cancer cells, in which one is prokaryotic, and another is eukaryotic. There may be a biocompatibility issue in long-term usage.	Stimulated the maturation of dendritic cells in lymph nodes to activate the immune response against melanoma	[44]
7.	Erythrocytes/Cancer cells. The copper sulfide nanoparticles were prepared by a solvothermal method using CuCl_2_ as a precursor and PVPK-30. The whole blood from BALB/c nude mice was centrifuged to remove the platelets, plasma, and WBCs, and suspended in normal saline (NS). For RBC lysis, deionized water was added and centrifuged to remove the hemoglobin several times, and the RBC membrane was retained. B16-F10 cell membrane was extracted by protein extraction kit after harvesting the cells with a cell scraper. To prepare RBC-B16 hybrid membrane, RBC membrane was mixed with B16-F10 membrane at different ratios and sonicated for membrane fusion. CuS NPs solution was added at different concentrations to the fused RBC-B16 hybrid membrane and sonicated to prepare the final product of CuS@[RBC-B16]. The excess unbound membrane was removed by centrifugation.	Copper sulfide nanoparticles	-	Doxorubicin	Melanoma	Photothermal/Chemotherapy. By fusing RBCs and B16-F10 cell membranes, they created a hybrid RBC-B16 biomimetic coating that can be coated onto hollow copper sulfide nanoparticles and used in combination with doxorubicin (DOX)-loaded photothermal/chemotherapy. Combined with DOX’s high loading efficiency and CuSs’ inherent photothermal conversion property, DCuS@[RBC-B16] achieved outstanding results in synergistic photothermal/chemotherapy of melanoma in vivo due to its excellent immune evading and homogenous tumor targeting abilities. The limitation of this research is that the fused membrane is prepared from RBCs, which can have ethical concerns and scaling-up issues.	Specifically enhanced the prolonged circulation lifetime, homogeneous targeting abilities, and self-recognition of target cells	[45]
8.	Cancer cells/Bacterial vesicle. The eukaryotic–prokaryotic vesicle EPVs were engineered by the fusion of OMVs of Salmonella with CMVs of B16F10 melanoma cells. Salmonella OMVs were obtained by engineered centrifugation, and CMVs were obtained by hypotonic disruption of the cell membrane followed by differential centrifugation. For PTT, three types of vesicles were made using the poly(lactic-co-glycolic acid)–indocyanine green (ICG) moiety (PI) and only OMV, only CMV, and only EPV to obtain PI@OMV, PI@CMV, and PI@EPV, respectively.	Eukaryotic–prokaryotic vesicle	-	Poly(lactic-co-glycolic acid) PLGA nanoparticles/indocyanine green (ICG)	-	Therapeutic vaccine. The eukaryotic-prokaryotic vesicle (EPV) is constructed by implanting outer membrane vesicles (OMVs) into cancer cell membrane vesicles (CMVs) as a tumor-specific antigenic nanoplatform with self-adjuvanting activities. Besides an efficient DC-based immunoactivation, the hybrid fusion vesicle confers robust CTL-derived immunity that is tumor-specific. It is possible to boost immunity against cancer cells by vaccinating with such an integrated EPV. In vivo studies showed its efficacy against melanoma management. In addition, EPV nano vaccines are capable of carrying therapeutic payloads for synergistic treatments for PTT and immunotherapy. The limitation of this study lies in the hybridization of eukaryotic and prokaryotic membranes, which have altogether different characteristics.	Potent prophylactic cancer vaccine	[46]
9.	Cancer cells/Dendritic Cells. Briefly, cancerous 4T1 cells and DCs were mixed at a ratio of 1:2 and were fused using DMSO and cultured in RPMI 1640 containing IL-4 for eliciting sufficient expression of pMHC as well as co-stimulatory molecules on the cell membrane. The fused cell membrane was extracted by the extrusion method. Using porphyrin-based Zr-metal−organic frameworks (PCN-224), PCN@ FM was synthesized by cloaking PCN-224 over FM using ultrasound in an ice bath. Following the same protocol, DC cell membrane-coated PCN@DM, and 4T1 cell membrane-coated PCN@CM, were prepared.	-	-	Zirconium-based MOFs	Breast cancer	Immunotherapy. By engineering tumor-specific immune-nanoplatforms using biologically derived fused cells (FC) cytomembranes (FM), they intend to facilitate easy cooperation with traditional nanotherapy. In this study, FCs were produced by fusing dendritic cells with murine mammary carcinoma tumors (4T1). Most of the interfacial characteristics of the two parent cells would be inherited by the FM-coated nanoparticles (NP@FMs), including specific targeting of homologous tumors, lymph node homing, the inclusion of tumor antigens, and co-stimulatory molecules to enhance the immune response. The nanotherapeutic could provide durable immunotherapy for the primary tumors in a tumor-bearing mouse post-PDT treatment after intravenous administration. The limitation of this study is the use of heavy metal-based nanoparticles, such as zirconia, which may impart hepatotoxicity.	Created immune response in tumor-bearing mouse model and inhibited the rebound of primary tumors by inducing photodynamic activity	[47]
10.	Cancer cells/Dendritic cells. Murine mammary carcinoma (4T1) cells were fused using PEG with the dendritic cells and the fused cells were cultured in RPMI 1640 for further 6 days for the production of pMHC, co-stimulatory molecules, lymph node homing receptors (C–C chemokine receptor type 7, CCR7). The fused cell membrane was isolated by lysis through repeated freezing and thawing, followed by the extrusion method of membrane isolation. Zirconia-based MOF was used to cloak the fused cell membrane.	-	-	-	Multiple tumor types	Nanovaccines. The cytomembranes of fused cells (FCs) of dendritic cells (DCs) and tumor cells were utilized to engineer biologically derived tumor-specific vaccines. To provide nanosized vaccines (NP@FM), nanoparticles were incorporated as the supporter of FMs in consideration of NPs’ well-known merits, such as their long circulating duration and passive targeting of tumors. The limitation of this study is the use of heavy metal-based nanoparticles, such as zirconia, which may impart hepatotoxicity.	Mimics tumor cells and functions like antigen-presenting cells	[48]
11.	Macrophage/Cancer cells. The cell membranes were isolated individually from RAW cells and 4T1 cells by repeated centrifugation, lysis, and extrusion method. Later the two individually isolated membranes were fused by sonication to obtain the fused membrane (FM). Doxorubicin (Dox) was added to PLGA, and to it, the different membranes were added individually for coating by sonication. The fused membrane-cloaked nanoparticles were named DPLGA@[RAW-4T1] NPs.	PLGA nanoparticles	-	Doxorubicin	Breast cancer	Cancer therapy. A hybrid membrane-coated doxorubicin (Dox)-loaded poly(lactic-co-glycolic acid) nanoparticle (DPLGA@[RAW-4T1] NPs) was synthesized by fusing membrane components derived from RAW264.7 (RAW) and 4T1 cells (4T1). Breast cancer metastases were treated with these NPs. Breast cancer with lung metastases was successfully treated with synthesized DPLGA@[RAW-4T1] nanoparticles, which resulted in prolonged survival without over-cardiotoxicity. The limitation of this study lies in the use of doxorubicin, which may impart cardiotoxicity in long-term usage.	Facilitated anti-metastatic treatment and prolonged the survival rate	[49]
12.	Erythrocytes/Platelets. RBC membranes were fused with platelet membranes and the fused membrane was extracted and coated over preformed poly(lactic-co-glycolic acid) (PLGA) core nanoparticles by sonication method to yield [RBC-P]NPs.	PLGA nanoparticles	Green fluorescent dye and red fluorescent dye	-	-	Enhanced hybrid functionalities. In the resulting hybrid cell membrane-coated nanoparticles, proteins from each source cell are retained and their unique functions are combined. A biocompatible nanocarrier with increasing complexity can be fabricated using the approach described in the research publication. The limitation of this research is that the fused membrane is prepared from cells of human origin, which can have ethical concerns and scaling-up issues.	Produced nanoparticles with advanced characteristics	[50]

**Table 3:** Nanodecoys in theranostic applications.

S. No	Cell-Membrane	Nanoparticles	Outcome	References
1.	Macrophages	Silver nanoclusters	The wavelength dependence of silver nanoclusters and the therapeutic agent made them the best suitable theranostic agent in fluorescence imaging	[61]
2.	Cancer cells	Gold NPs and platinum skin cores	Cell membrane of the formulation functioned as a cancer-cell recognition tool, and the core acted as the signal transducer	[74]
3.	Cancer cells (Brain tumor cells)	Lanthanide-doped nanoparticles	Prepared nanoprobe was used for an imaging brain tumor and to achieve precise diagnosis during the surgery	[75]
4.	Cancer cells	Citric-stabilized-ultrasmall iron oxide nanoparticles (USIO NPs)	The particle designed was pH-responsive and performed ultrasound-enhanced MRI-guided delivery of doxorubicin, thereby providing image-guided chemotherapy.	[76]
5.	Myeloid-derived suppressor cell membrane	Pristine magnetite nanoparticles	The formulation actively targeted tumor to provide immune evasion, MR imaging, and photothermal therapy against tumor cells.	[77]

**Table 4:** Nanodecoys for vaccination in cancer immunotherapy.

S. No	Cell-Membrane	Nanoparticle	Surface Modifications	Drug	Outcome	References
1.	Cancer cells	-	PEGylation	-	Exhibited high serum stability and draining efficiency to local lymph nodes	[81]
2.	Cancer cells	Aluminum phosphate nanoparticles	-	Cytosine-phosphate-guanine (CpG) oligonucleotides	Suppressed tumor growth and prolonged survival of mouse model	[82]
3.	Cancer cells		-	Functional DNA, CpG oligonucleotide, and aptamer	Improved therapeutic responses and elimination of the majority of the tumors; provided long-term immunity	[83]
4.	Erythrocytes	PLGA nanoparticles	Mannose	-	Demonstrated great potential in cancer immunotherapy	[84]
5.	Cancer Cell	Adjuvant Nanoparticles	Mannose		Possessed great efficacy in delaying tumor development as a prevention vaccine	[85]
6.	Bacterial cell	-	-	PC7A/CpG polyplex core	Facilitates in situ immune recognition and enables a novel personalized approach	[86]
7.	Cancer cell (Acute myeloid leukemia cell membrane)	PLGA nanoparticles		CpG adjuvant	Activated AML-specific immune responses and provided a long-term anti-leukemic survival benefit	[87]

## Data Availability

Not applicable.
